# Predicting common bile duct stones: Comparison of SAGES, ASGE and ESGE criteria for accuracy

**DOI:** 10.12669/pjms.38.8.6666

**Published:** 2022

**Authors:** Muhammad Nadeem Yousaf, Yasir Mahmud, Shahid Sarwar, Muhammad Nauman Ahmad, Mahmood Ahmad, Ghulam Abbas

**Affiliations:** 1 Dr. Muhammad Nadeem Yousaf, MBBS, FCPS (Med). Senior Registrar, Allama Iqbal Medical College Lahore/Jinnah Hospital Lahore, Lahore - Pakistan; 2 Dr. Yasir Mahmud, MBBS, FCPS (Med). Consultant Physician, Allama Iqbal Medical College Lahore/Jinnah Hospital Lahore, Lahore - Pakistan; 3 Dr. Shahid Sarwar, MBBS, FCPS (Med), FCPS (Gastroenterol), MCPS-HPE, FRCP (Edin). Professor of Medicine, Allama Iqbal Medical College Lahore/Jinnah Hospital Lahore, Lahore - Pakistan; 4 Dr. Muhammad Nauman Ahmad, MBBS. Post graduate Resident, Allama Iqbal Medical College Lahore/Jinnah Hospital Lahore, Lahore - Pakistan; 5 Dr. Mahmood Ahmad, MBBS, FCPS (Gastroenterol). Assistant Professor Gastroenterologist, Allama Iqbal Medical College Lahore/Jinnah Hospital Lahore, Lahore - Pakistan; 6 Dr. Ghulam Abbas, MBBS, FCPS (Gastroenterol). Assistant Professor Gastroenterology, Allama Iqbal Medical College Lahore/Jinnah Hospital Lahore, Lahore - Pakistan

**Keywords:** ASGE, Common bile duct stones, ERCP, ESGE, Risk stratification, SAGES

## Abstract

**Objective:**

To determine accuracy of SAGES, ASGE and ESGE criteria for predicting presence of common bile duct (CBD) stones.

**Methods:**

In a prospective study at Jinnah Hospital Lahore from March 2021 to February 2022, patients with suspected CBD stone were stratified in High risk (HR), intermediate risk (IR) and low risk (LR) for SAGES, ASGE and ESGE criteria. All patients underwent ERCP and risk strata were analyzed using SPSS 22® for sensitivity, specificity, positive predictive value (PPV), negative predictive value (NPV) and accuracy.

**Results:**

In 90 patients with mean age 45.18(±14.87) and male/female ratio 0.64(35/55), area Under Curve (AUC) for predicting CBD stones was 0.75, 0.81and 0.83 for HR and 0.28, 0.52 and 0.52 for IR group while it was 0.53, 0.81 and 0.53 for absence of stone in LR group of SAGES, ASGE and ESGE criteria respectively. HR groups had accuracy of 81.1%, 86.7% and 87.8% in predicting CBD stone while LR criteria had 68.8%, 86.7% and 68.1% accuracy in predicting absence of CBD stone for SAGES, ASGE and ESGE respectively.

**Conclusion:**

HR strata of SAGES, ASGE and ESGE scores have excellent accuracy in predicting CBD stones whereas IR and LR criteria are suboptimal for excluding CBD stones.

## INTRODUCTION

Common bile duct stones are among leading causes of extra hepatic cholestatic jaundice. Approximately 10-20% patients with gall stones have stones in CBD as well.^1^ Many of these patients remain asymptomatic for years however they may develop recurrent right sided abdominal pain or in some cases ascending cholangitis and acute pancreatitis may complicate cholidocholithiasis.

Stones in CBD warrant immediate removal via endoscopic retrograde cholangiopancreaticography (ERCP) as per American Society of Gastrointestinal Endoscopy (ASGE) guidelines.^2^ ERCP has transformed the management of CBD stones and now we can handle even difficult large size stones with large balloon papillary sphincteroplasty, cholangioscopy guided intraductal laser or electrohydaulic lithotripsy.^3^ However 15-25% of ERCPs being performed for CBD stones fails to identify any stone despite using best available diagnostic modalities like MRCP or endoscopic ultrasound before planning ERCP.^4^ Being an invasive procedure, 5-15% patients undergoing ERCP can develop complications like pancreatitis (16.4% in a study of 165 patients)^5^, bleeding (1.34%)^6^ or perforation (0.6%).^6^ Therefore selection of patients for ERCP needs to be done carefully to avoid unnecessary intervention.

In order to restrict ERCP to only patients with highest probability of actually having stone in CBD, we need accurate and reproducible risk stratification strategies.^7^ This risk stratification should be based on simple, economical and easily available diagnostic tools like clinical data, liver function tests, abdominal ultrasound etc. However, accuracy of these scoring system should be high to reduce number of unnecessary ERCPs without missing patients with CBD stones.

Number of scoring systems have been proposed recently by different international societies for predicting presence of cholidocholithaisis before listing patients for ERCP. American Society for Gastrointestinal Endoscopy (ASGE) proposed a scoring system in 2010 which has recently been modified in 2019 for risk stratifying patients with CBD stones.^2^ Similarly Society of American Gastrointestinal and Endoscopic Surgeons (SAGES) and European Society of Gastrointestinal Endoscopy (ESGE) have proposed their own scoring systems for predicting likelihood of cholidocholithasis before proceeding for ERCP.^8^,^9^ These Scores are based on presence of jaundice, dilated common bile duct on ultrasound, visible stone in CBD on imaging and presence of ascending cholangitis to stratify patients in high risk (HR), intermediate risk (IR) or low risk (LR) categories.

Despite being used in clinical practice for selecting patients for ERCP, very few studies have evaluated these scoring systems for accuracy. Pretest probability of SAGES criteria in predicting CBD stones varies between 50-90%, 5-50% and < 5% for HR, IR and LR groups respectively as depicted in literature while another study noted pre-test probability of >50%, 10-50% and <10% for ASGE criteria for high, intermediate and low risk groups respectively.^10^ However more data regarding predictive accuracy of these risk stratification scores is needed to establish their place in clinical practice. It will enable us to apply most accurate score while selecting patients for ERCP thus avoiding need for expensive tests like MRCP and CT scan without missing patients with CBD stones. We planned a study to determine the diagnostic accuracy of SAGES, ASGE and ESGE criteria for predicting presence of CBD stone.

## METHODS

Study was carried out at Department of Gastroenterology Jinnah Hospital Lahore from March 2021 to February 2022. Sample size of 80 was calculated with confidence level 95%, margin of error < 5% and expected accuracy of 70% for risk stratification scores. After approval by Ethical Review Board of Institution (Ref No 70^th^/ERB dated 17^th^ March 2021), consecutive patients undergoing ERCP for extraction of CBD stones were included in study. We included patients with CBD stone on ultrasound abdomen, deranged LFTs with common bile duct more than 6 mm on ultrasound and no identifiable cause of biliary obstruction, ascending cholangitis with CBD >6mm or deranged LFTs and clinical suspicion of extra-hepatic biliary obstruction on imaging. We excluded patients with biliary pancreatitis, biliary stricture or stenosis, ampullary cancers, cholangiocarcinoma, liver cirrhosis, viral hepatitis, chronic alcoholism and those who had previous surgical or endoscopic interventions like cholecystectomy, ERCP with sphincterotomy, pancreatico-biliary or gastric surgeries.

Detailed clinical history and examination was performed on each patient and based on clinical evaluation and laboratory and radiological investigations including complete blood count, liver function tests and abdominal ultrasound, three risk stratification scores, ASGE, ESGE and SAGES were calculated for each patient. The SAGES criterion had four risk factors; common bile duct stone (CBDS) on imaging, dilated common bile duct (CBD), ascending cholangitis, and total bilirubin (TB) > 1.7 mg/dL. Patients with ≥ 2, 1, and 0 factors were stratified as HR, IR, and LR, respectively. In ASGE criterion, patients with CBDS on imaging, ascending cholangitis, or TB > 4 mg/dL plus dilated CBD were stratified as HR. The IR criteria included abnormal liver function tests (LFTs), age > 55 year, or dilated CBD, and LR if none of these risk factors were present. In ESGE risk stratification we classified patients as HR (had cholangitis or CBDS on imaging), IR (had abnormal LFTs or dilated CBD), and LR (none of these risk factors were present). CBD size more than 6mm on ultrasound was considered as dilated. Presence of echogenic focus with acoustic shadow in CBD was considered as evidence of CBD stone on imaging. Values of bilirubin, ALT, AST or ALP more than reference range were labelled as deranged LFTs. Ascending cholangitis was diagnosed on presence of Charcot’s triad of fever, jaundice and abdominal pain.

All patients underwent ERCP at Department of Gastroenterology Jinnah Hospital Lahore. Presence of CBD stone was verified on stone extraction during procedure. In case of failure to extract stone, presence of floating negative shadow on fluoroscopic image of CBD during cholangiography was also diagnosed as CBD stone. ERCPs where selective CBD cannulation could not be done were excluded from final analysis. Patients were monitored for post-ERCP complication after inpatient admission for 24 hours.

### Statistical Analysis:

Data was analyzed using SPSS version 22® (Armonk NY: IBM Corp.). Quantitative variable like age, duration of illness, liver function tests, CBD size etc. were expressed as mean ± standard deviation (SD), whereas, qualitative variables like gender, presence of CBD stone on imaging, ascending cholangitis, stone extraction on ERCP etc. were given in percentage.

For risk stratification scores of SAGES, ASGE and ESGE, we calculated Area Under Curve (AUC) and then determined sensitivity, specificity, positive predictive value, negative predictive value and accuracy of high risk(HR), intermediate risk (IR) and low risk (LR) categories of ASGE ESGE and ASGES for predicting presence or absence of stone. P value of ≤ 0.05 was considered significant for all statistical analyses.

## RESULTS

Total of 90 patients were included in study. Mean age of included patients was 45.18±14.87 years with male to female ratio of 0.64(35/55). Jaundice was the most common presenting complaint in 54(60%) patients, 39(43.3%) had itching, 21(23.3%) had fever while clay colored stools were present in 14(15.6%) patients. Hypertension was noted in 12(13.3%) patients and four (4.4%) were diabetic.

On ultrasound examination, CBD stones were identifiable in 66(77.3%) patients while 24(26.7%) had no visible CBD stone on ultrasound. CBD was more than 6mm in 77(85.6%) patients, 70(77.8%) of whom had dilated intra-hepatic ducts as well. Stone in gall bladder was identified in 70(77.8%) patients while three (3.3%) patients already had cholecystectomy. Ascending cholangitis was diagnosed in 11(12.2%) patients. Magnetic resonance cholangiopancreaticography (MRCP) was performed in 13 patients due to non-conclusive ultrasound and all had CBD stone on it.

All patients underwent ERCP electively. CBD was dilated in 79(87.8%) patients on ERCP and CBD stones were identified on cholangiogram in 60(66.7%) patients while in 30(33.3%) no stone was retrieved. Biliary stenting was done in 20(22.2%) patients. Color of retrieved stones was yellow in 40(44.4%) patients while it was pigmented black in 20(22.2%) patients. All patients had uneventful recovery except four (4.4%) patients who had post-ERCP pancreatitis with recovery after hospital admission.

We applied SAGES, ASGE and ESGE risk stratification to all included patients. In SAGES risk staging of included patients, 69(76.7%) were high risk, 19(21.1%) were intermediate risk and two (2.2%) patients were in low risk category, on applying ASGE staging, 68(75.6%) were in HR, 87(96.7%) in IR and 22(24.4%) were in LR strata whereas 67(74.4%) patients were in high risk strata of ESGE, 87(96.7%) in intermediate risk and 2(2.2%) in low risk criteria of ESGE. Area Under Curve (AUC) for predicting presence of CBD stone on ERCP for high risk (HR) patients was 0.75(95% confidence interval (CI)-0.63-0.86), 0.81(95% CI-0.70-0.92) and 0.83(95% CI-0.72-0.93) for SAGES, ASGE and ESGE respectively. (Graph-I) For intermediate risk (IR) categories of SAGES, ASGE and ESGE, AUC for predicting presence of CBD stones was 0.28(95% CI-0.16-0.40), 0.52 (95% CI-0.39-0.65) and 0.52(95% CI-0.39-0.65) respectively which are suboptimal. AUC for low risk (LR) patients for predicting absence of CBD stone on ERCP was 0.53, 0.81 and 0.53 for LR group of SAGES, ASGE and ESGE criteria respectively. (Graph-II) In view of suboptimal AUC for IR category in all three scores, we decided to restrict accuracy analysis to HR category for presence of stone and LR strata for absence of CBD stone on ERCP.

**Graph-I:**
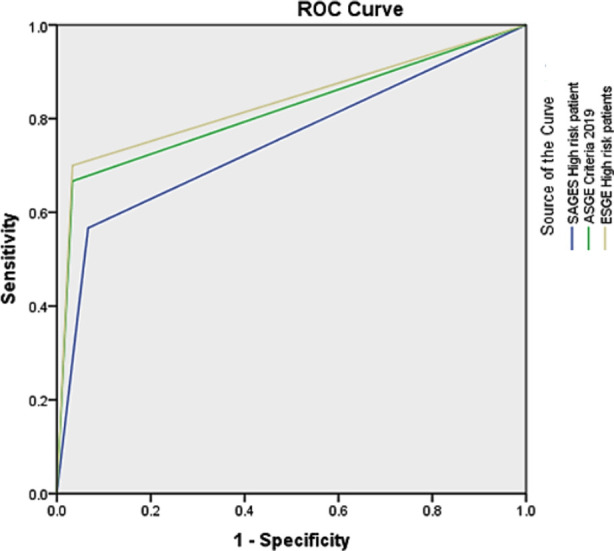
ROC curve and AUC values for High Risk groups for predicting presence of CBD stone.

**Table:** Area Under the Curve

Test Result Variable(s)	Area
SAGES High risk patient	.750
ASGE Criteria 2019	.817
ESGE High risk patients	.833

**Graph-II:**
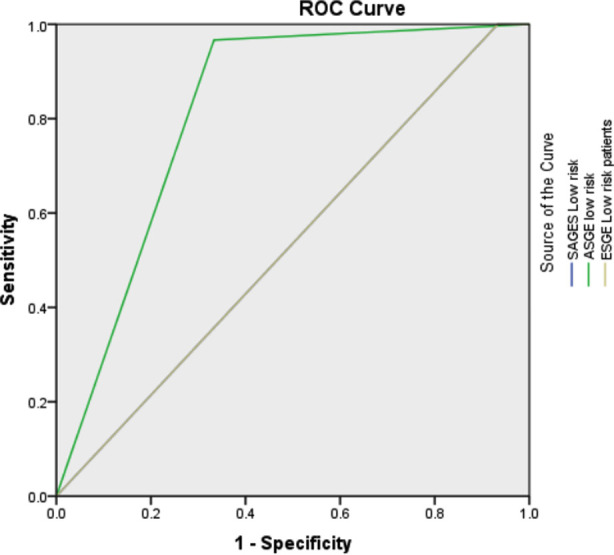
ROC curve and AUC values for low risk groups for predicting absence of CBD stones.

**Table:** Area Under the Curve

Test Result Variable(s)	Area
SAGES Low risk	.533
ASGE low risk	.817
ESGE Low risk patients	.533

High risk groups in SAGES, ASGE and ESGE scoring systems have shown accuracy of 81.1%, 86.7% and 87.8% in predicting presence of CBD stone on ERCP. Sensitivity, specificity, positive predictive value (PPV) and negative predictive value (NPV) are given in Table-I. We evaluated accuracy of low risk groups of these scoring systems for predicting absence of CBD stone which was 68.8%, 86.7% and 68.1% for SAGES, ASGE and ESGE respectively. Detailed accuracy analysis of low risk group is shown in Table-II.

**Table-I:** Accuracy of high risk (HR) group for predicting presence of CBD stones.

Score	Sensitivity (%)	Specificity (%)	PPV (%)	NPV (%)	Accuracy (%)	P value
SAGES	93.3 (56/60)	56.6 (17/30)	81.1 (56/69)	80.9(17/21)	81.1(73/90)	<0.0001
ASGE	96.6 (58/60)	66.7 (20/30)	85.2(58/68)	90.9 (20/22)	86.7 (78/90)	< 0.0001
ESGE	96.7(58/60)	70 (21/30)	86.5 (58/67)	91.3 (21/23)	87.8 (79/90)	<0.0001

**Table-II:** Accuracy of low risk (LR) group for predicting absence of CBD stones.

Score	Sensitivity (%)	Specificity (%)	PPV (%)	NPV (%)	Accuracy (%)	P value
SAGES	6.7 (2/30)	100 (60/60)	100 (2/2)	68.1(60/88)	68.8(62/90)	0.04
ASGE	66.7 (20/30)	96.7 (58/60)	90.9 (20/22)	85.2 (58/68)	86.7 (78/90)	< 0.0001
ESGE	6.7% (2/30)	100 (60/60)	100 (2/2)	68.1 (60/88)	68.1 (62/90)	0.04

## DISCUSSION

Stone in common bile duct is not always visible on imaging studies especially in distal CBD due to its retro-peritoneal location and overlapping intestinal loops. Diagnostic accuracy of ultrasound is also operator dependent and varies between 20-80% for CBD stones.^11^ Different hepatobiliary professional societies have recommended use of risk stratification criteria for selecting patients for retrieval of CBD stones on ERCP. We have compared accuracy of SAGES, ASGE and ESGE criteria in predicting presence or absence of CBD stones.

In our study, high risk (HR) group of all three criteria have shown excellent AUC of 0.75, 0.81 and 0.83 for SAGES, ASGE and ESGE respectively. In a study of 280 patients by Wangchuk K et al, AUC for SAGES, ASGE and ESGE were 0.77, 0.75 and 0.74 respectively.^12^ The diagnostic accuracy of HR group for CBD stone was 81.1% (Sensitivity 93.3% specificity 56.6%), 86.7%(sensitivity 96.6% specificity 66.7%) and 87.8%(sensitivity 96.7% specificity 70%) for SAGES, ASGE and ESGE respectively in our study. It was 78.9% (81.1% sensitivity, 72.06% specificity), 75% (75.4% sensitivity, 73.5% specificity) and 70%(66.04% sensitivity, 82.35% specificity) for SAGES, ASGE and ESGE HR groups in study of Wangchuk K et al.^12^

HR group criteria of all three scoring systems in our study met the requirement of >50% accuracy in HR group, as defined by ASGE Standards of practice Committee.^13^ Sensitivity of 96.6% of HR groups of both ASGE and ESGE criteria in our study is better than 74.64% and 73.64% for ASGE and ESGE respectively identified in a study by Jagtap N et al.^14^ However, we noted specificity of 66.7% and 70% for HR group of ASGE and ESGE which is less than what Jagtap N et al noted, 74.2% and 82.35% and Reddy et al noted, 83.4% and 87.3% for HR groups of ASGE and ESGE scoring respectively.^15^ In a study of 179 patients by Adams MA et al, overall accuracy of high risk strata of ASGE criteria was 62.1% with sensitivity of 47.4% and specificity of 73.7%.^16^

On comparing low risk (LR) criteria of three scoring systems for predicting absence of CBD stone in our study, ASGE LR strata has much better accuracy of 86.7%(sensitivity 66.7%, specificity 96.7%) as compared to LR groups of ASGE and ESGE, 68.8% and 68.1% respectively. In a study of 267 patients comparing accuracy of ASGE criteria 2010 with that of 2019, 83% patients were rightly selected for ERCP with 2019 criteria as compared to 79% with 2010 ASGE criteria.^17^

Despite optimal performance of these risk stratification scores in correctly identifying patients with CBD stones as per ASGE standard of practice committee, we still are likely to have negative ERCPs while applying these criteria. Area of concern is lower specificity of around 60-70% of these scores whereas sensitivity is more than 93% therefore if we use these criteria for selecting patients for ERCP, we are unlikely to miss a patient with CBD stone but will have around 30% ERCPs with no CBD stones. High specificity and low sensitivity of LR criteria for absence of stones will also have same implication, patients without CBD stones not meeting LR criteria are likely to be exposed to unnecessary intervention. With 5-15% chances of serious complications like perforation, bleeding and pancreatitis with ERCP, ability of these risk stratification criteria to exclude patients with no CBD stones still needs improvement.

With availability of non-invasive tests like Endoscopic ultrasound (EUS) which has sensitivity of 95% and specificity of 97% and MRCP with 93% and 96% sensitivity and specificity respectively for correctly identifying CBD stones,^18^ approach in selecting patients for ERCP is fast evolving. However, limited availability of these advanced diagnostic tools, high dependence on operator’s expertise, risk of sedation, bleeding and perforation in EUS and their high cost warrants further research to refine and improve performance of risk stratification scores.^19^

### Limitations of the study:

We included patients from one tertiary care center limiting generalizability of our data. We have presented data of 90 patients, larger sample size would have overcome patient related confounding variables like age, gender ethnicity etc. We have not analyzed intermediate risk (IR) group further due to suboptimal AUC on ROC analysis limiting utility of this strata for selecting patients for ERCP. Despite these limitations, our study is first from this region to evaluate performance of these simple risk stratification criteria for selecting patients for retrieval of CBD stones with ERCP.

## CONCLUSION

High risk strata of SAGES, ASGE and ESGE scores have excellent accuracy in predicting presence of CBD stones however further refinement of these scores is needed to avoid negative ERCPs. Patients with intermediate or low risk scores need further testing with EUS or MRCP before deciding need for ERCP.

### Author’s contribution:

**MNY:** Data collection, revising the manuscript.

**YM:** Conception, data collection, revising the manuscript

**SS:** Conception, Design, analysis and interpretation, drafting of article

**MNA:** Analysis of data, revising manuscript critically.

**MA:** Data collection, revising manuscript critically.

**GA:** Data collection, revising manuscript critically.

All authors have approved the final version and are accountable for the integrity of the study.
